# Survey of Communicable Diseases Surveillance System in Hospitals of Iran: A Qualitative Approach

**DOI:** 10.5539/gjhs.v8n9p44

**Published:** 2015-10-16

**Authors:** Nayeb Fadaei Dehcheshmeh, Mohammad Arab, Abbas Rahimi Foroushani, Fereshteh Farzianpour

**Affiliations:** 1 Public health school, Tehran University of Medical Sciences, Tehran, Iran; 2 Health Economics and Management Department, Tehran University of Medical Sciences, Tehran, Iran; 3 Statistics and epidemiology Department, Tehran University of Medical Sciences, Tehran, Iran

**Keywords:** hospital, disease reporting, communicable diseases, surveillance system

## Abstract

**Background:**

Communicable Disease Surveillance and reporting is one of the key elements to combat against diseases and their control. Fast and timely recognition of communicable diseases can be helpful in controlling of epidemics. One of the main sources of management of communicable diseases reporting is hospitals that collect communicable diseases’ reports and send them to health authorities. One of the focal problems and challenges in this regard is incomplete and imprecise reports from hospitals. In this study, while examining the implementation processes of the communicable diseases surveillance in hospitals, non-medical people who were related to the program have been studied by a qualitative approach.

**Methods:**

This study was conducted using qualitative content analysis method. Participants in the study included 36 informants, managers, experts associated with health and surveillance of communicable diseases that were selected using targeted sampling and with diverse backgrounds and work experience (different experiences in primary health surveillance and treatment, Ministry levels, university staff and operations (hospitals and health centers) and sampling was continued until arrive to data saturation.

**Results:**

Interviews were analyzed after the elimination of duplicate codes and integration of them. Finally, 73 codes were acquired and categorized in 6 major themes and 21 levels. The main themes included: policy making and planning, development of resources, organizing, collaboration and participation, surveillance process, and monitoring and evaluation of the surveillance system. In point of interviewees, attention to these themes is necessary to develop effective and efficient surveillance system for communicable diseases.

**Conclusion:**

Surveillance system in hospitals is important in developing proper macro - policies in health sector, adoption of health related decisions and preventive plans appropriate to the existing situation. Compilation, changing, improving, monitoring and continuous updating of surveillance systems can play a significant role in its efficiency and effectiveness. In the meantime, policy makers’ and senior managers’ support in development and implementation of communicable disease surveillance’ plans and their reporting plays a key and core role.

## 1. Introduction

Nowadays, the communicable disease control is announced as one of the most important health issues, at the international level, to prevent the spread of the diseases (Kebede et al., 2011; Rutherford, 1998). Trying for early revealing and controlling of communicable diseases, the increase of new emerging and re-emerging communicable diseases, including Drug-resistant TB, SARS and Ebola, as well as the increasing of the need for information in health system have led to more attention to the communicable diseases surveillance system (Jelastopulu, Merekoulias, & Alexopoulos, 2010; Perry et al., 2007).

Regardless of tremendous advances of medical sciences in the areas of prevention and treatment of communicable diseases, unfortunately, a communicable disease with the potential capacity to cause epidemics is still a public health problem throughout the world. The incidence of drug resistant pathogens and their vectors (Malaria, Gonorrhea, Meningitis, & Bacterial pneumonia), returning some of diseases to areas that for years have been free of disease (Tuberculosis & Malaria) and the appearance of new diseases (Ebola, Avian influenza, AIDS, & SARS), all of are the reasons for involved sectors’ attention to them. Vital parts of public health programs in each country are, surveillance of communicable diseases in order to prevent and control, identify the prevalence and burden of diseases, accomplishment of interventions, assess the effectiveness of surveillance programs, management and health resource allocation (Keramarou, & Evans, 2012; Yoo et al., 2009).

Some believe that, in developing countries, diseases surveillance has get new form and gradually the burden of communicable diseases has been diminished, non-communicable diseases has been replaced, while the reality isn’t that. The burden of communicable and infectious diseases hasn’t been reduced; instead, it has been increasing during the last years. Some behavioral diseases such as HIV, hepatitis and sexually transmitted diseases have been added. On the other hand, many infectious diseases have left their previous geographic position and reached other regions of the world; Cholera, malaria, dengue fever, Rift Valley fever and other viral blood drop fever are some of these types of diseases. However, besides them, the incidence and prevalence of novel infectious diseases and controlled infectious disease has been reemerged progressively too (Guya Mohammad, 2009). Communicable diseases threat public health increasingly and they have imperative role in growing health surveillance costs in many countries, especially developing countries. Information of surveillance can be used for policy-making, planning, implementation, resource allocation, forecasting and early discovery of epidemics at local, national and international level (Azar, Masoori, Meidani, & Paul, 2010). To reduce the burden of communicable diseases, especially in developing countries, data production should be strengthened by the creation of precise and possible surveillance to this situation and their risk factors. In many parts of the world patient surveillance has been committed and has been recognized as one of the significant components of prevention and control programs (Tan, Chang, Tseng, & Lin, 2007).

Reporting is the base of surveillance in health system. It is considered as a crucial point in all health policy makings in countries and the information is considered as a health priority (Ashraf Khorramirad, & Zahra Moharrami, 2011). Communicable disease reporting is a vital element of planning and evaluation of prevention and control programs (Marzieh Nojumy, 2003).

Because of the importance and impact of communicable diseases, as well as their diverse distribution, regulations in national and international level for communicable disease surveillance and reporting have been designed and described (Yoo et al., 2009; Hanafusa et al., 2012; Reintjes, Thelen, & Reiche, 2007). In Iran, similar to other countries, the purposes of surveillance and reporting system, list of diseases and the type of reporting, reporting sources and legal obligation of reporting have been identified (Tabatabai, Ahmad nia, GHotbi, & Rahimi, 2006).

Accurate and timely notification by reporting sources for quick and timely response is essential to increase the incidence of disease. Epidemiological characteristics such as incidence may affect timeliness in reporting, which can be facilitated by familiarity with the disease in the population. The different patterns of time lags among the diseases examined suggest that there are common features among the disease groups, and strategies for better control should be developed specifically for each disease group (Yoo et al., 2009). Therefore, respond to an epidemics depends on the ability of the surveillance system and reporting of diseases to provide information for actions timely. Diseases reporting can be done by health centers, hospitals, clinics, private clinics and laboratories, but, unfortunately, there are some reporting problems in these centers (Jelastopulu, Merekoulias, & Alexopoulos, 2010; Perry et al., 2007; Keramarou & Evans, 2012; Tabatabai, Ahmad nia, GHotbi, & Rahimi, 2006; Tandir, Sivic, Tandir, & Zunic, 2011).

In spite of the undeniable importance and necessity of reporting on disease control and surveillance, statistics of not reported is high by physicians and many studies in Germany, Australia and Turkey indicate physicians, who worked in primary health surveillance systems has insufficient information in the list of diseases and reporting requirements (Tandir, Sivic, Tandir, & Zunic, 2011). In Redid and Beckerman’ study, which was conducted in Saudi Arabia (2000), the rate of diseases reporting is 74 percent (Bakarman, & Al-Raddadi, 2000). In another study conducted in the United States, only 55% of physicians and 63 percent of nurses in emergency departments and primary health surveillance centers had adequate knowledge about reporting diseases (Turnberg, Daniell, & Duchin, 2010).

The hospital is one of the cornerstones of the health system of any country and it is dominant in the control and management of diseases. One of the most important tools for disease management in hospitals is communicable disease surveillance and reporting system. It collects the reports of communicable diseases to record on surveillance system, pursuing incidences and control actions and sends them to related health authorities in the province level (Tabatabai et al., 2006).

Communicable diseases Surveillance system in hospitals varies depending on the types of diseases. Diseases that are subject to surveillance and reporting in hospitals are divided in two groups: diseases subject to immediate reporting and non-urgent reporting. Observing immediate reporting diseases, they must be reported to hospital’s communicable disease connector, by telephone, as soon as possible and within 24 hours. Diseases such as acute flaccid paralysis, diphtheria, congenital rubella, neonatal’ and adults’ tetanus and malaria, etc. are part of this group. For reporting of non-urgent diseases, when suspected case is observed, in addition to inform connector, personalized patient assessment form must be completed and according to cases, they must be sent to related authorities by fax weekly or monthly. Diseases such as smear negative pulmonary tuberculosis, sexually transmitted diseases, brucellosis, anthrax, etc. are part of this category (Qolahi, 2013).

Unfortunately, one of the challenges in this respect is incomplete and wrong reports and in some cases the reports less than the hospitals’ actual amount (Shinde et al., 2009). Accomplished studies about physicians’ knowledge in care system in Iran are different and variable from 14.3% to 38%. About reporting diseases a study in Shiraz showed in 6 months only 7.4% from physicians had reported a disease. Another study showed only 18.5% physicians had reported in a year. Registered statistics during a year in three health centers in Tehran were less than one percent (Kolahi, 2013).

For these reasons, the need for study in the area of communicable disease surveillance system in hospitals was felt. In few studies that have been done in the area of disease surveillance, they concentrated more to physicians’ attitudes. Present study, while examining executive processes of communicable diseases surveillance, non-medical related people to program also have been studied by a qualitative approach.

## 2. Methods

This Study was conducted using qualitative approach and content analysis technique from December 2014 up to April 2015.

Participants in the study included 36 informants, managers, experts associated with health and surveillance of communicable Diseases that were selected using targeted sampling and with diverse backgrounds and work experience (different experiences in primary health surveillance and treatment, Ministry levels, university staff and operations (hospitals and health centers) and sampling was continued until arrive to data saturation. Selection criteria for entering were at least one year of experience related to the communicable diseases surveillance system in the hospital and expression of those experiences. Nine (9) of the participants were female and 27 were male, their ages were between 30-70 years with a mean of 50, their work experience was between 6-30 years with mean of 18 years.

For data collection, semi-structured depth interviews were conducted. After obtaining informed and oral consent from the participants and explained the objectives of the study, face to face interviews were done in agreed time in participants’ workplace. The interviews were recorded by a digital recorder. They took between 75-30 minutes.

Immediately after finishing of each interview, the texts were typed and reviewed several times. Then, meaning units were identified and initial codes were extracted. In the following, codes are integrated and then, classified according to similarities. Finally, the concept and hide content of data was taken out.

Guidance questions for interview were as following:

1) How does the Current trend of Communicable Diseases Surveillance in the general hospitals affiliated to University of Medical Sciences?
2) What are the problems and obstacles of Communicable Diseases Surveillance in the general hospitals affiliated to University of Medical Sciences?
3) Which factors influence Communicable Diseases Surveillance in the public hospital affiliated to University of Medical Sciences?
4) What are the solutions to improve performance of Communicable Diseases Surveillance in the general hospitals affiliated to University of Medical Sciences?

The interview was directed based on the participants’ response. During the interview, while articulating their experiences, the researcher controlled his accurate perception from participants’ words by helping questions. The next questions were raised regarding to the participants’ experiences.

Qualitative content analysis was used to analyze the data. In this way, by systematic classification, the codes and themes are identified.

To assess the data and ensure the accuracy and reliability of data, four factors, acceptance, affiliation or reliability, verification and transfer capability according to Lincoln’ and Gobi’ theory (quoted from Shenton) were used. For that, the researcher had long-term relationship (15 years) with research places that helped increasing participants’ confidence and understanding the study environment better. Participants’ review was used to confirm the accuracy of the data and codes. In fact, after encoding the interview, texts were returned to the participants to ensure about the accuracy and interpretations of the codes and the codes that were not show the participants’ opinion were modified. Also, it tried to choose diverse participants in terms of age, specialty, gender and workplace. The interviews were reviewed by outside observers familiar with qualitative research. The Codes and classified levels were given to several faculty members to be examined. For transmissibility of findings, it was tried to present participants’ quotes exactly.

## 3. Results

In analyzing the interviews, after elimination of duplicated codes and integration of similar cases, 73 codes were acquired, that were classified in 6 major themes and 21 levels (Table 1). They will be explained separately.

**Table 1:** Themes and levels

Theme	Level
Policy making and planning of surveillance system	• Development of Laws, regulations and policies of surveillance system
	• Fascinating the Support and positive outlook of policymakers and chief managers of treatment area
	• Compilation of Joint Executive Guidelines of surveillance system (Health)
	• Compilation of strategic plan for surveillance system of university level

Development of the resources of Surveillance system	• Compensation of employees
	• Development of human resources and specialist of program
	• Designing website (Software) for data of surveillance System based on early warning system

Organizing the surveillance system	• appropriate organizational structure of surveillance system
	• coordination committees and meetings of surveillance system

Cooperation and coordination surveillance system	• strengthening inter-sectorial and multi-sectorial cooperation (primary health and treatment)
	• Appealing the confidence and participation of health staff
	• Mutual respect and understanding among staff of primary health deputy and deputy of treatment

The process of surveillance	• physician’ diagnosis
	• Documents registration
	• Reporting to primary health sector
	• Receive feedback from health center’s performance

Monitoring and evaluation of the surveillance system	• Designing reward and punishment system for surveillance system
	• Deputy of primary health and deputy of treatment monitoring implementation
	• The position of surveillance system in the accreditation of hospital
	• Determination of the performance indicators of the surveillance system

### 3.1 Policy Making and Planning of Surveillance System

This theme includes four levels as development of laws, regulations and surveillance policies, support and positive outlook of policy makers and managers of treatment area, developing joint surveillance’ operational guidelines (health) and strategic planning of the surveillance system.

1-1, Development of Laws, regulations and policies of the surveillance system.

It is necessary to collect sufficient data from existing diseases in community, to estimate the population health status, appropriate planning of health services and evaluation of their quality. Surveillance system is the foundation of diseases control in health surveillance system. It is as a key point in all health policy makings in different countries.

“Reporting of communicable diseases is so old law that has not been updated for many years. It is necessary this law be revised by the Commission of Health in parliament that, hospital’s treatment system becomes actually accountable.

“One of the essential issues can be traced back to the policy making. The first law approved for diseases reporting system came back to many years ago. The fine that parliament has considered for this program is too small, so, the legislation and revising debate have specific sensitivity.”

1-2, Fascinating the Support and positive outlook of policymakers and chief managers of treatment area.

Since, deputy of health in Ministry of Health has fundamental responsibility for Communicable Diseases surveillance in Iran, its implementation in hospitals doesn’t have operational and staff managers’ support of treatment area. Attraction the support of policy makers and chief managers of treatment sector and their positive attitude will have key role in the successful implementation of the program. Some participants believed that, this program isn’t the priority of treatment area, and they are not concerned in this regard.

“In treatment area, the attitude towards reporting is weak and in Ministry level, they don’t know how importance this reporting.”

“In addition to difference Vision in treatment area, even those accept the importance of primary health services, have other priorities”.

1-3, Compilation of Joint Executive Guidelines of surveillance system (Health)

Due to mentioned issues and examination of other challenges from experts’ and participants’ perspective in the interviews, compilation of guidelines and executive joint regulations of health using brainstorming and acquired experts’ opinions in operational and academic levels has specific importance and can contribute to the promotion and implementation of the program in Iran’s hospitals. (This is done correctly, if it is imparted by deputy of treatment for implementation in hospitals obligatorily).

“It is necessary that experts in both primary health and treatment deputies who also have worked at the operational level, with an expertise action and surveys of academic experts write joint guideline”

“As long as we are not coordinated each other at ministerial level and we don’t have common guideline, we won’t have it in public environment. Therefore, everybody will think their benefits”.

1-4, Compilation of strategic plan for surveillance system of university level

Hospitals in medical university level are under treatment deputy of the university. Performing of the program in hospitals faced with some challenges such as gaps between different Deputies. Because of that, compilation plan for surveillance system is necessary. Strategic planning of university which carried out under direct supervision of the President of the University should require all deputies (primary health, treatment, education and research development) to support the program and it will be the concern of the whole system. Respectively, the amount of responsibility and accountability, as well as interaction and cooperation will permeate to lower levels.

“I believe that, surveillance system is the base of our all planning and it should be placed in strategic level of universities. In this fashion, not only the Deputies of primary health and treatment, health centers and hospitals, but also deputies of culture & students and research & education will involve and support the program. If we reach to unity in our opinions and procedures, we can join all sectors by strategic planning”.

“Once, the deputy of treatment had not involved in discussion of pregnant women’ death and they categorized it as responsibility of primary health deputy. However, when work was began and meetings were held, treatment sector noticed, and then, the system of surveillance for pregnant women’ death in hospitals was established. As a result, they practiced the responsibility that diverse sectors must have to this issue. This is a good experience for Communicable Diseases Surveillance at the macro level of the university”.

### 3.2 Development of the Resources of Surveillance System

This theme contains three main categories of compensation of employees, human resources and professional development and design of the website (software) of surveillance system’s data based on early warning system.

#### 3.2.1 Compensation of Employees

Most of participants believed that payment to employees in fee for service form of surveillance system program has impacts on promote motivation, increased commitment, and improved of performance. On the other hand, operation of surveillance system to pay for physicians, nurses and other involved forces can affect the program. In the absence of cooperation in the program, it should be considered this in paying for them. Performance-based incentive system will be particularly important in the program.

“Infection control nurse does not have any financial support, and this tends to nurses’ discourage.”

“Incentive system in hospital’s surveillance system is not defined. In WHO standards, when a certain guideline is introduced, the first thing to be considered is an incentive system for employees.”

#### 3.2.2 Development of Human Resources and Specialist of Program

Human resource was the main factor that most participants stated. Human education and training was one of the frequent codes in interviews. Many participants believed that, the deputy of education should change educational Curriculum of medical and nursing students in regards to program carrying out. In their internship, Communicable diseases surveillance system emphasizes more. In addition to academic education, training at the beginning of employment, training during work years, holding educational workshops to change attitudes and insights of employees and enhance their knowledge and skills is crucial.

“A person, who is in charge of program in hospital, must be convinced by training classes and essential educations.”

“One of the discussions is related to training. I was a medical student. Something was thought us, we study and take a test, but there are other things that you need to practice. Surveillance system is one of those needs to have a special position in internship. “

#### 3.2.3 Designing Website (Software) for Data of Surveillance System Based on Early Warning System

Experts and managers in the study believed that, one of the weaknesses of the program in hospitals is unavailability of comprehensive networking system. Unfortunately, despite the hospitals have health information system (HIS); the surveillance system is not included on HIS. A comprehensive network is needed in health surveillance system which includes Communicable Diseases Surveillance program too. On the other hand, this software should be designed based on the early warning system and be able to report outbreaks and epidemics in smart way to related authorities.

“One of the problems is that, despite the implementation of IHR, in contrast to developed countries, there is no online system to register and report diseases at the moment.”

“One problem is that hospitals have purchased HIS from different companies, so they are not standardized. I hope the HIS system is linked to SEPAS (Persian Electronic Health Records network).”

### 3.3 Organizing the Surveillance System

This theme includes two main categories; appropriate organizational structure and meetings and committees of the coordination in Surveillance system.

#### 3.3.1 Appropriate Organizational Structure of Surveillance System

This study showed that, the structure of health surveillance system in the country contributes to the implementation of the program. In some provinces and cities of Iran, the hospitals according to the organizational and official hierarchy are separated from health system structure and placed directly under the Deputy of treatment (It should be noted that in the capitals of provinces, because there are no primary health network, health centers are under deputy of primary health and hospitals are under deputy of treatment). Therefore, in these cases, the level of cooperation and coordination seems lower than provinces and cities that hospitals and health centers are under health network and they have integrated and have unique management.

The results showed that, because there are numerous Universities of Medical Sciences in Tehran, sometimes “incoordination and structural problems in the area of health may exist. The hospitals of a university for implementing of certain health programs such as surveillance program have to communicate with other universities’ health centers that it causes creation of some problems in implementation phase.

“In many provinces that the hospitals are under health network, there is more interaction. But, elsewhere, the hospitals structurally separated from health network, cooperation is low and there are a series of problems”.

“A main problem in big cities like Tehran is that, a number of University of Tehran’s hospitals send their reports to west health district which is under Iran University and a vicious cycle has been created.”

Other factor related to the organizational structure which mentioned frequently by participants was the structure of human resource. What was clear in this study is that, the surveillance program doesn’t have specific authority in the hospitals, and at the universities, different people and units take responsibility for the program (Expert on environmental health, infection control nurse, educational supervisors, matron, and temporary deployment force in health centers). According to the survey, the majority of authorities in hospitals and the deputy of treatment complained about shortage of treatment workers and claimed that, city’s health center is responsible for the implementation of this program and the hospitals don’t have any responsibility for that. The main mission of the hospital is treating patients and their satisfaction.

“Public hospitals affiliated to universities have an outdated structure and a number of organizational posts which are assigned in unacceptable norm. It is inconsistent with the facts, and this causes a lack of employees in our hospitals.”

“As to the forces involved in the program are not fixed and they experience constantly changing workplace, this movement hurts to accomplishment of the program.”

#### 3.3.2 Coordination Committees and Meetings of Surveillance System

Communicable disease surveillance program in hospitals is the multi-sectorial joint activity in the health system. According to participant’s views, meetings and joint committees in the deputy and city levels to examination of the problems and recommendation of solutions will help the improvement of the quality and performance of the program. In addition, the organizing of Communicable Diseases Surveillance committee in hospitals with contribution of the manager and units’ authorities (inpatient and outpatient) impacts on suitable implementation of the program.

“I think there is a lack of cooperation and coordination between primary health surveillance and treatment” their relationship isn’t close. “

“Several factors affect the program. One is that, the primary health services are not coordinated with treatment part. Coordination in higher level should be taken.”

### 3.4 Cooperation and Coordination Surveillance System

This theme includes 4 main categories; multi-sectorial and inter-sectorial cooperation within (primary health and treatment), strengthening medical staff’ beliefs, fascination confidence and participation of health workers and mutual respect and understanding of primary health and treatment parts’ staff.

#### 3.4.1 Strengthening Inter-Sectorial and Multi-Sectorial Cooperation (Primary Health and Treatment)

Participants in the study acknowledged that, in regards with the goals of surveillance system and increase the productivity of attempts, it should be increased the cooperation among the various sectors such as hospitals’ outpatient departments (clinics, professional clinics and emergence departments) and inpatient departments which emphasize more on potent cooperation among matron, supervisors, and nurse’s office, and control of infection. An important issue that mentioned in interviews was that, according to the physicians’ vital role in the surveillance system, especially in clinics and emergency department, their cooperation is necessary.

“Nurses occupations don’t allow them to have upright cooperation. Clinics’ Cooperation is really weak. If the departments were close to each other, it was better and I think the productivity became much better. “

“One of the problems in surveillance system of hospitals is that, physicians do not cooperate and the nurses are busy.”

Another factor that was important in point of participants in the research is holding and strengthens cooperation and communication between primary health and treatment in different levels of Ministry of Health, Universities and operating.

“The thing is clear that, there are no cooperation and coordination between the Deputy of Primary Health and Deputy of Treatment in any levels.”

#### 3.4.2 Appealing the Confidence and Participation of Health Staff

Implementation of Communicable Diseases Surveillance programs in the hospitals without considering the role of primary health sector isn’t possible. Participation of health experts in direction and guidance of hospitals in this field is undeniable. It is worth to say that, as the information and reports of hospital surveillance program should be sent to the health centers, Primary health experts’ confidence and beliefs to hospitals have a crucial role in the progress and success of the program.

“The importance of this issue for all must be cleared and a truthful atmosphere must be formed between primary health workers and hospital.”

“Current surveillance system is not valid. Primary health sector have a little belief and trust to reports and Statistics of hospitals, and it is necessary to increase primary health’ trust to the performances of the program in the hospital.”

#### 3.4.3 Mutual Respect and Understanding among Staff of Primary Health Deputy and Deputy of Treatment

Since the program has the common components and points between the primary health and treatment sectors, there are differences of opinions and styles between these two sectors according to the results. One reasons has been stressed by participants was lack of knowledge and awareness of primary health workers about the problems and challenges of treatment area and hospitals. Hospitals said that health centers judge by their own views and they don’t consider priorities and limitations of hospitals. But what clear is that, with respect and mutual understanding can be achieved to an agreement and empathy in this area.

“We want more respect from primary health experts. They think that problems are only for them, while we have our own limitations.”

“Naturally primary health and treatment must be closed, know their positions, and respect each other’s. There are some biases: treatment sector says primary health surveillance just make disturbance and they always want reports, or, primary health workers say treatment sector don’t believe to preventive health surveillance and they don’t have preventive vision. These thoughts must be eliminated. “

### 3.5 The Process of Surveillance

This theme includes four main categories; physician’s diagnosis, documentation, reporting to health centers and receiving performance feedback from primary health sector.

#### 3.5.1 Physician’ Diagnosis

According to the results, the first step of surveillance system is timely and correct diagnosis of physicians. This diagnosis in hospital’s surveillance system affected by factors such as physicians’ attitudes, the amount of knowledge and information, practical skills, experience and history of work in health sector, healthy orientation, income, the number visits and performance feedback to physicians.

“The most important thing is early diagnosis of physicians. We can implement the program according to their diagnosis. In fact, if diseases are not diagnosed, we can’t do any action.”

“Physicians have complained that they do not have additional time, they just write a prescription and refer to the nurse fast.”

“The physicians are trained seven years to treat and they have treatment vision. They aren’t health-orientation. They have not been trained for this program.”

#### 3.5.2 Documents Registration

Another important component of the surveillance process is properly registration of information related to the program. Participants in the study believed that, the registration system in hospitals doesn’t work rightly. Medical records and patients’ records are as important documents to check the status surveillance system. It is believed that, this program should be placed as the part of physician’s order and supervisors’ reports.

“The registration system does not work correctly, record and forms filled incompletely.”

“The role of medical evidence in documentation and tracking of cases is very vital.”

#### 3.5.3 Reporting to Primary Health

One of duties of hospitals in surveillance system is reporting cases require follow-up to health center of city. Selection of responsible person for the reporting and the way of reporting are effective factors.

According to experts’ opinion, the lack of coordination between primary health surveillance and treatment, and the lack of reporting the cases and incomplete reports are noticeable problems.

“One of the major challenges of surveillance system is call and report cases to the health centers. In fact, nobody report cases or the reporting done with delay.”

“In this case, coordination between hospitals and health centers is poor. Reporting is not upright, and some reports are also very incomplete.”

#### 3.5.4 Receive Feedback from Health Center’s Performance

The results showed that, receiving performance feedback and feedback of reported cases from primary health sector has influence on workers’ motivation and improving hospitals’ performance.

Impact of analysis and hospitals’ performance was done by primary health experts to guide and reduce the error rate were the issues that the participants stressed.

“One thing that is important and I’ve experienced is that, every three months, the primary health sector give feedback to hospitals. Providing feedback to the hospitals is very effective.”

“Provision of Feedback by health centers to hospitals is effective to increase the interest and motivation of the personnel. Usually, feedback from primary health sector to hospitals isn’t done. “

### 3.6 Monitoring and Evaluation of the Surveillance System

This theme includes six main categories; determine performance indicators of surveillance system, supervision of deputy of primary health and treatment, the hospital surveillance system’ position in hospital accreditation, and designing reward and punishment system of surveillance system.

#### 3.6.1 Determine the Performance Indicators of the Surveillance System

Some participants in the study stressed that the expectations and goals of the surveillance system should be defined in terms of performance indicators. And these expected indicators are sent to all parts of the hospital.

“It is necessary that the surveillance system is introduced as an indicator of hospital, and the results are supplied for all.”

“Actions of health surveillance system should be sited as one of the performance indicators of treatment area (The rate of hospitals’ cooperation in surveillance system must be determine as an indicator of performance).”

#### 3.6.2 Deputy of Primary Health and Deputy of Treatment Monitoring Implementation

Interviewees stated that, in order to implement the guidelines and protocols of surveillance system, identifying problems and challenges of the program, and reforming of the processes in order to improve program performance, it needs deputy of primary health and deputy of treatment jointly monitor hospital performance at the Ministry and the university level.

“Monitoring how the surveillance program works should be the part of deputies of primary health’s and treatments’ monitoring program, and to identify the problems of health program it must be conducted mutually.”

“It should be designed common inspection of two deputies in the form of common check list to monitor and provide feedback to meet deficiencies.”

#### 3.6.3 The Position of Surveillance System in the Accreditation of Hospital

Performance indicators of the surveillance system should be considered in hospital accreditation process and rated high degree that hospital managers and staff understand the importance of the program.

“The surveillance system program should be considered in evaluation of hospital. For example, it is considered in hospital accreditation program and assigned high score to have influence.”

According to the current status, as long as the surveillance system is not considered in preferences of hospital authority, its importance won’t be determined. It is necessary to be included in the accreditation of hospitals and allocate good score for that.

#### 3.6.4 Designing Reward and Punishment System for Surveillance System

Most participants noted that, the surveillance system needs to have an incentive system based on reward and punishment arrangement. Strengthen the motivation of interested people and dealing with those who fail in their duties, can become suitable tools for better implementation of the program.

“Effective factors are returned to human resources that are really imperative. Incentives have important role that can be defined rewards in financially mode. “

“There is nothing to create commitment. If we can design a type of motivation system, it can be effective in the implementation of the program.”

## 4. Discussion

The aim of this study was to make out obstacles and problems of hospitals’ surveillance system, identifying factors impacting the management of communicable diseases surveillance, and providing ways to improve its performance.

The results of this study showed that, for having effective communicable diseases surveillance system in hospitals, some of the areas must be noticed such as policy making and planning, resource development, organization, collaboration and participation, monitoring process and evaluation of surveillance programs.

Reporting system and diseases surveillance system has fragile infrastructure and laws in most countries. Also, they don’t systematically use data of communicable disease surveillance system to prioritize issues and compilation of programs (Shinde, Kembhavi, Kuwatada, & Khandednath, 2012; Miller, Deeble, Roche, & Spencer, 2004; Sahal, Reintjes, Mahgoub, & Aro, 2011). Because of that, governments should revise the laws, regulations and policies which relate to this program and create inter-sectorial and multi-sectorial collaboration to management of the program, in which, all private and public sectors involve. Using appropriate technologies for this program, governments reinforce disease surveillance systems (Perry et al., 2007; Sahal, Reintjes, Mahgoub, & Aro, 2011; Sahal, Reintjes, Eltayeb, & Aro, 2011). In this study, the absence of macro policy-making in the field of communicable diseases surveillance, no revision in old rules, a lack of collaboration between two deputies of the Ministry of Health, and the lack of comprehensive surveillance programs in the universities from the points of experts, mentioned as problems and shortcomings of the surveillance system in Iran. Therefore, an effective and efficient surveillance system in hospitals requires macro policy-making and planning in the Ministry of Health and the university level.

Development of information infrastructure and human resources is another factor that can influence the performance of a communicable disease surveillance system. The best communicable disease surveillance system and reporting must identify incidence and prevalence of diseases quickly and respond to disease outbreaks timely and appropriately. Therefore, completeness and timeliness of the data is a key element in creating the surveillance system in hospitals (Reintjes, Thelen, & Reiche, 2007; Haq & Khan, 2013; Vogt, Spittle, Cronquist, & Patnaik, 2006). Because of resources lack, the problems of using paper based methods and conquer on inefficiencies in information transformation, the need to utilization of electronic, simple and efficient methods to surveillance and reporting of communicable diseases is felt (Rajeev et al., 2010; Rumisha, Mboera, Senkoro, Gueye, & Mmbuji, 2007). Slowness of non-electronic reporting system would make it difficult to analyze the information (Nnebue, Onwasigwe, Ibeh, & Adogu, 2013). Development of electronic systems can be used as quick, efficient, effective and affordable tools for this purpose (Hanafusa et al., 2012; Turner, Reeder, & Ramey, 2013). In this study, the expert believed that, one of the weaknesses of the hospital surveillance system is the unavailability of a comprehensive electronic network in health system. However, the application of an electronic system for this purpose needs funding resources and budget to establish the system, inter-sectorial cooperation, personnel training and efforts to maintain the confidentiality of patient information (Faensen et al., 2006; Sickbert-Bennett, Weber, Poole, MacDonald, & Maillard, 2011).

In this study, from the perspective of professional, the employees play a key role in providing qualified services in surveillance of diseases. Resources shortages and high workloads can affect people’s motivation to work. In some cases, standard processes for communicable disease surveillance and reporting from low level to high level is not available or it is complex and has little flexibility against changes (Shinde, Kembhavi, Kuwatada, & Khandednath, 2012; Miller, Deeble, Roche, & Spencer, 2004). One of the main rings in the system of surveillance and reporting of communicable disease is physicians. Studies show that, time consuming of surveillance and reporting of communicable diseases’ form, unfamiliarity of doctors with the relevant forms, lack of adequate notification on this issue, insufficient knowledge of physicians about the list of disease in surveillance area and reporting the performance of program are influential factors (Qolahi, 2013; Nader & Askarian, 2009; Sahal, Reintjes, Eltayeb, & Aro, 2010).

Identify specific processes and standards, but, simple in surveillance and reporting of communicable disease, assignment of the person responsible for these affairs, consider the moral and material incentives to individuals, can have main role in improvement of diseases surveillance process. Insufficient education, lack of skills and knowledge in the field of communicable disease surveillance and reporting, produced serious obstacles against public health programs (Staes et al., 2009; Nader & Askarian, 2009; Nnebue, Onwasigwe, Adogu, & Onyeonoro, 2012; Tan, Yeh, H-W. Chang, C-K. Chang, & Tseng, 2009).

Develop clear job duties for clinical staff of hospitals and the person who is responsible for communicable disease reporting, as well as training courses in cooperation with the various sectors of health system can be effective to eliminate these obstacles and problems (Perry et al., 2007; Yoo et al., 2009; Tan, Chang, Tseng, & Lin, 2007; Hanafusa et al., 2012; Dixon, Grannis, & Revere, 2013; Karami & Abedini, 2012). In Iran, even though, there is a general guideline for surveillance and reporting of communicable diseases, but in the case of hospitals, which have unique properties, it isn’t considered distinct guideline and followed the same general guidelines.

The existence of an appropriate organizational structure can play a role in communicable disease surveillance and reporting. Defining Systematic structure by specifying the duties of individuals and joint meetings between the primary health authorities and hospital could improve the surveillance and reporting of communicable diseases. As several studies have pointed out that the lack of a clear framework in this area and the shortage of understanding between the primary health connectors and hospitals authorities causes deterioration in their relations. This impacts the quality of surveillance and reporting and lessens sensitivity of the issue for hospital staff. Lack of clarity of need for reporting, feeling of not using of reports, undetermined the identity of the recipient and others are leaflets factors that impact management of surveillance and reporting. The majority of these issues can be solved by developing a suitable organizational structure and inter-sectorial and multi-sectorial coordination (Hanafusa et al., 2012; Qolahi, 2013; Dixon, Grannis, & Revere, 2013).

There are several problems in the field of monitoring and evaluation of activities related to communicable diseases surveillance from the perspective of surveillance experts. Communicable disease surveillance and reporting system in hospitals does not follow the specific guidelines for monitoring and evaluation. However, several studies have emphasized their existence (Vavalle, 2010; Bino et al., 2013; Karami & Abedini, 2012; Djibuti, Rukhadze, Hotchkiss, Eisele, & Silvestre, 2007). The evaluation plan of communicable diseases surveillance should be constructed and implemented in hospitals with the participation of various primary health and treatment sectors. A good system of surveillance must have appropriate monitoring and evaluation plan that can discover the problems of communicable disease surveillance system and reporting quickly and take action to resolve them (Shinde et al., 2012; Staes et al., 2009; Sahal, Reintjes, Mahgoub, & Aro, 2011; Bino et al., 2013; Sickbert-Bennett, Weber, Poole, MacDonald, & Maillard, 2011). In this area by setting performance indicators for surveillance and reporting of communicable diseases, design a supervisory system based on encouragement, determine the position of communicable disease surveillance and reporting in the form of accreditation standards and strict hospitals to careful implementation of surveillance and not reporting more or less than usual confidence.

## 5. Conclusions

Surveillance system in hospital has an important role in disease management. For proper management of communicable diseases control, comprehensive and updated information is required. Surveillance system of communicable diseases is important in developing appropriate macro policies of health system, adoption of health decisions and accurate preventive actions to the existed situation.

Given that, because of incorporation between primary health and treatment sectors, inefficient resources management, sectorial-orientation, education sector’s challenges, staff’s low motivation, weak supervision, the surveillance system in Iran suffers some problems and defects. In addition, there are no separate guidelines in surveillance and reporting of communicable diseases in Iran’s Hospital. Thus, it is possible to design surveillance system and reporting of hospitals with consideration of physical and software infrastructure, and the assistance and cooperation of other sectors of health system. It should be noted that the change, improvement, continuous monitoring and updating of surveillance systems can play a significant role in its efficiency and effectiveness. To acquire these goals, in first step, policy makers’ and senior managers’ support seems to be necessary. Convinced that, the surveillance of communicable diseases has a significant impact in promoting the health of the community, can play an effective role in the development and implementation of these programs accurately. The involvement of medical universities in the designing and execution of surveillance plans and reporting of communicable diseases as well as imposing the private sector to conform to these standards, can improve surveillance and reporting processes of communicable diseases.

**Study limitations**

- The coordination with managers was time-consuming.
- In the treatment area there wasn’t certain responsible units in this regard and it was different in each university.
- Internal Materials and documents in this field was very low
- Because the topic was common in the area of primary and treatment of health system, coordination and data collection was difficult.
